# Long-Lasting Effect of Infant Rats Endotoxemia on Heat Shock Protein 60 in the Pancreatic Acinar Cells: Involvement of Toll-Like Receptor 4

**DOI:** 10.1155/2012/354904

**Published:** 2012-05-22

**Authors:** Joanna Bonior, Jolanta Jaworek, Michalina Kot, Stanisław J. Konturek, Piotr Pierzchalski

**Affiliations:** ^1^Department of Medical Physiology, Faculty of Health Sciences, School of Medicine, Jagiellonian University, Michalowskiego 12 Street, 31-126 Krakow, Poland; ^2^Physiology Medical Faculty, School of Medicine, Jagiellonian University, Grzegorzecka 16 Street, 31-531 Krakow, Poland

## Abstract

*Introduction*. Lipopolysaccharide endotoxin (LPS) is responsible for septic shock and multiorgan failure, but pretreatment of rats with low doses of LPS reduced pancreatic acute damage. *Aim*. We investigated the effects of the endotoxemia induced in the early period of life on Toll-like receptor 4 (TLR4), heat shock protein 60 (HSP60) and proapoptotic Bax, caspase-9 and -3 or antiapoptotic Bcl-2 protein expression in the pancreatic acinar cells of adult animals. *Material and Methods*. Newborn rats (25 g) were injected with endotoxin (*Escherichia coli*) for 5 consecutive days. Two months later, pancreatic acinar cells were isolated from all groups of animals and subjected to caerulein stimulation (10^−8^ M). Protein expression was assessed employing Western blot. For detection of apoptosis we have employed DNA fragmentation ladder assay. *Results*. Preconditioning of newborn rats with LPS increased TLR4, Caspase-9 and -3 levels, but failed to affect basal expression of HSP60, Bax, and Bcl-2. Subsequent caerulein stimulation increased TLR4, Bcl-2, and caspases, but diminished HSP60 and Bax proteins in pancreatic acinar cells. Endotoxemia dose-dependently increased TLR4, Bax, HSP60, and both caspases protein signals in the pancreatic acini, further inhibiting antiapoptotic Bcl-2. *Conclusions*. Endotoxemia promoted the induction of HSP60 *via* TLR4 in the infant rats and participated in the LPS-dependent pancreatic tissue protection against acute damage.

## 1. Introduction

Lipopolysaccharide (LPS, endotoxin), which is a constituent of the outer membrane of gram-negative bacteria, plays a very important role in the pathogenesis of septic shock [1]. LPS is the pivotal stimulus for triggering an inflammatory cascade in macrophages *via* Toll-like receptor 4 (TLR4). LPS has also been identified as a ligand for TLR4 and takes a part in the pathophysiology of the sepsis syndrome [2–4]. Several pathways of endotoxin signal transduction have been suggested in case of endotoxin stimulation of the cell.

Toll-like receptors (TLRs), originally identified as homologues of *Drosophila *Toll, belong to the superfamily of interleukin-1 receptors [5]. TLRs are the most important family of pattern recognition receptors (PRRs) [6, 7]. The existence of several TLRs enables the innate immunity system to recognize different groups of pathogens while initiating appropriate and distinct immunological responses, according to the pathogen-associated molecular patterns (PAMPs) [8]. TLR4 proteins are expressed on the cell surface becoming the receptors for the Gram-negative bacteria cell membrane components, LPS. Stimulation of TLR4 by LPS is a complex process, which includes the participation of several molecules like LPS binding protein (LBP), CD14, and MD-2 [9, 10]. TLR4 might participate in the induction of both protective and harmful effects on the tissues. Beside TLR4, other ligands of TLR4, like hyaluronan, induce an immunological response initiating epithelia repair but in some cases TLR4 are involved in conveying of an endogenous danger signals mobilizing high-mobility group box-1 protein or in response to free fatty acids what results in tissue damage [11–13]. Some reports point out the possibility of TRLR4 involvement in response to heat shock protein 60 (HSP60) as an endogenous ligand TLR4 [14].

HSP60 is involved in the protein folding, assembly, disassembly, and degradation under normal conditions. This protein, similar to other HSPs, is increased during cellular stress as an adaptive protection strategy [15]. Over the past decade investigators found that HSP60 and the pancreatic enzymes share a common location inside the pancreatic acinar cells, interacting intimately [16, 17]. Moreover, like the distributive characteristics of pancreatic enzymes, HSP60 showed an increasing gradient of collocation along the pancreatic secretory pathway from the rough endoplasmic reticulum and Golgi apparatus to zymogen granules in the acinar cells [16]. An increased transcription and production of HSP60 with protective action has been suggested in pancreatitis [18–20].

Acute pancreatitis (AP) is an emergent disease commonly seen in the clinical practice but its complicated pathogenesis is still incomprehensible. Scientists are in agreement that AP involves a cascade of events, and numerous reports have suggested that its initial step is the activation of trypsinogen inside the pancreatic acinar cells, resulting in damages evoked by the activated pancreatic enzyme [21, 22]. As to the mechanism of the abnormal enzyme activation, a number of theories have been considered, for instance: calcium overload or cathepsin B activation [23, 24]. A new theory advocates that HSP60 plays an important role in the protection of pancreatic tissues against damages and malfunctioning or weakening of HSP60 effect under physiological conditions is responsible for the early zymogen activation in AP [15, 24].

It has been shown that low doses of endotoxin (LPS) could protect the pancreas against caerulein-induced pancreatitis (CIP) [25–28]. Endotoxemia in the suckling rats attenuates acute pancreatitis and impairment of the exocrine function *in vitro* and* in vivo* models at adult age [29–32].

The aim of this study was to investigate in the pancreatic acinar cells isolated from adult animals, the effects of foregoing infant rats endotoxemia on TLR4, HSP60 and pro-apoptotic Bax, caspase-9 and -3 or antiapoptotic Bcl-2 protein expression.

## 2. Material and Methods

Studies were performed on male Wistar rats (weighing: newborn 25 g; adult: 170–200 g). Animals were housed in cages under standard conditions, on commercial pellet chow at water *ad libitum*, at room temperature with a 12-h light and dark cycle.

### 2.1. Reagents

Lipopolysaccharide from Sigma-Aldrich Co. (St. Louis, MO, USA) and caerulein (Takus) from Pharmacia GmbH, Erlangen, Germany, were used for the experiments.

### 2.2. Experimental Protocol

The experimental protocol was divided into two general parts:* in vivo* and *in vitro *researches.

### 2.3. *In Vivo* Experiments

Newborn rats weighing 25 g were employed and divided into five main groups:

1. control group: rats were injected with 200 *μ*L of vehicle saline intraperitoneally (i.p.), once a day, during 5 consecutive days.
2. LPS (*Escherichia coli*) group: rats were treated with LPS dissolved in 200 *μ*L of vehicle saline, and animals were subjected to i.p. injection once a day, during 5 consecutive days. This rats were divided into three separate subgroups which were treated with a single dose of LPS:

2.1 group: 5 mg/kg/day × 5 days (total dose 25 mg/kg);

 2.2 group: 10 mg/kg/day × 5 days (total dose 50 mg/kg);

 2.3 group: 15 mg/kg/day × 5 days (total dose 75 mg/kg).

Each part of the study consists of several experimental groups of rats, 6–8 rats in each single group.

### 2.4. *In Vitro* Experiments

Two months following the injection of both vehicle saline or LPS solution, at adult age of animals, the pancreatic acinar cells were isolated by collagenase digestion as described previously [33, 34] and subjected to increasing concentration of caerulein (10^−12^, 10^−10^ or 10^−8 ^M). The cells were incubated in the presence of tested substance for: 0, 0.5, 1, 3, 5, or 7 h. Subsequently, 10^−8 ^M concentrations of caerulein were found to be the most effective (data not shown) and selected for further experiments. Time-course experiments have shown that 5 h incubation time was the most effective and has been picked out for all further part of the study (data not shown). All the experiments were repeated at last three times. The results presented here were taken from the most representative experiments.

All experimental procedures performed in this study were approved by the Jagiellonian University Ethical Committee for Animals Experimentation. 

### 2.5. Western Blot

The whole-cell extracts were prepared as described elsewhere [35]. Equal load of protein in each sample was assessed using QantiPro BCA Assay Kit (Sigma, USA). Protein samples were boiled with Western blot sample buffer and loaded on the 12% SDS-polyacrylamide gel. After electrophoresis and transfer of the samples, the PVDF membrane (BioRad, USA) was blocked with blocking buffer (5% non-fat dried milk in PBS) for 1 h in room temperature. Blocking procedure was followed with 1 h exposure to primary antibody diluted 1:1000 and secondary antibody diluted 1:1000 in blocking buffer.

 After each antibody probing membrane was washed three times for 15 min. in TBST buffer (0,1 M Tris pH 8,0; 1,5 M NaCl; 0,5% TritonX-100). Detection of membrane bound proteins was performed using BM Chemiluminescence Blotting Substance (Boehringer, Mannheim, Germany). The blots were stripped and probed with GAPDH to document equal protein loading. All presented results were obtained in 4 consecutive experiments and are representative for the observed phenomenon. The following items used in the Western blot reactions were purchased from Santa Cruz Biotechnology (Santa Cruz): antibodies mouse monoclonal anti-HSP60 IgG_1_[sc-136291], mouse monoclonal anti-caspase 9 IgG_1_ [sc-81663], mouse monoclonal anti-Bcl-2 IgG_1_ [sc-7382], mouse monoclonal anti-Bax IgG_1_ [sc-70408], mouse monoclonal anti-GADPH IgG_1_ [sc-137179], goat polyclonal anti-caspase 3 IgG [sc-1225], rabbit polyclonal anti-TLR4 IgG [sc-30002], rabbit anti-goat IgG HRP [sc-2768], and goat anti-mouse IgG_1_-HRP [sc-2060], goat anti-rabbit IgG-HRP [sc-2030].

### 2.6. DNA Fragmentation

To analyze DNA fragmentation due to induced apoptosis, cells (5 × 10^6^/sample) were lyzed with 150 *μ*L hypotonic lysis buffer (edetic acid 10 mM, 0.5% Triton X-100, Tris-HCl, pH 7.4) for 15 min. on ice and were precipitated with 2.5% polyethylene glycol and 1 M NaCl for 15 min. at 4°C. After centrifugation at 13000 × g for 10 min. at room temperature, the supernatant was treated with proteinase K (0.3 g/L) at 37°C for 1 h and precipitated with isopropanol at 20°C. Centrifuged pellets were dissolved in 10 *μ*L of Tris-EDTA (pH 7.6) and analyzed employing electrophoresis in a 1.5% agarose gel containing ethidium bromide. DNA pattern was visualized under ultraviolet light.

### 2.7. Statistical Analysis

All experiments were performed in triplicates. Results are expressed as means ± SEM. Statistical analysis was performed using analysis on variance and two-way ANOVA test when appropriate. Differences with *P* < 0.05 were considered as significant.

## 3. Results

*The Study of the Effects of Lipopolysaccharide (Escherichia coli) and/or Caerulein on TLR4, Bcl-2, Bax, HSP60, Caspase-9, and Caspase-3 Protein Level and Apoptosis in the PancreaticAcinar Cells.*

**Figure 1:**
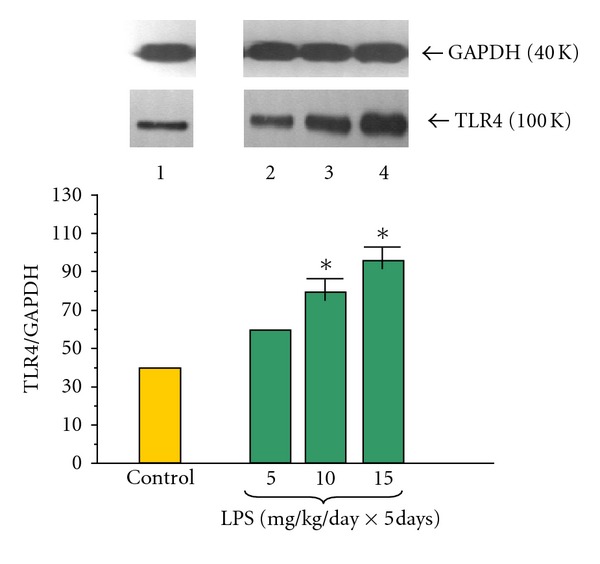
Western blot analysis of TLR4 protein level in the adult rat pancreatic acinar cells under basal conditions (lane 1), treated in the infant animals by lipopolysaccharide (*Escherichia coli*) at the doses of 5 mg/kg/day × 5 days (lane 2), 10 mg/kg/day × 5 days (lane 3), and 15 mg/kg/day × 5 days (lane 4) after 5 hours of incubation. Asterisk indicates significant (*P* < 0.05) change, as compared to the control group. The blots were stripped and probed with GAPDH to document equal protein loading. All presented results were obtained in 4 consecutive experiments and are representative for the observed phenomenon.

**Figure 2:**
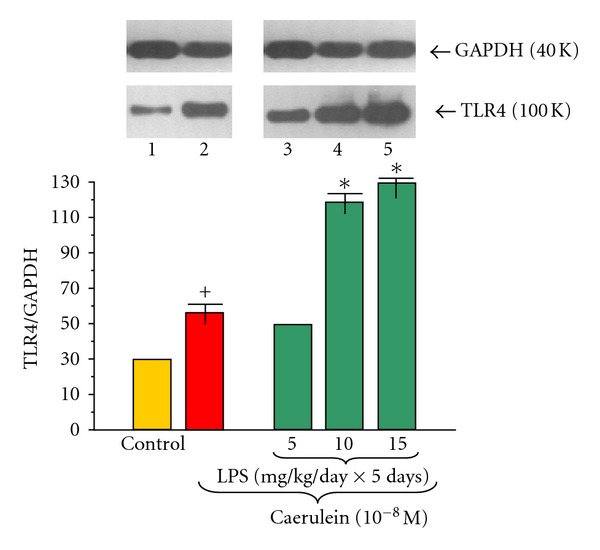
Western blot analysis of TLR4 protein level in the adult rat pancreatic acinar cells under basal conditions (lane 1), stimulated *in vitro* by caerulein at the dose of 10^−8 ^M (lane 2), treated in the infant animals by lipopolysaccharide (*Escherichia coli*) at the doses of 5 mg/kg/day × 5 days + caerulein 10^−8 ^M (lane 3), 10 mg/kg/day × 5 days + caerulein 10^−8 ^M (lane 4), and 15 mg/kg/day × 5 days + caerulein 10^−8 ^M (lane 5) after 5 hours of incubation. Cross indicates significant (*P* < 0.05) change, as compared to the control group. Asterisk indicates significant (*P* < 0.05) change, as compared to the value obtained from the rats treated by increasing doses of LPS (10 or 15 mg/kg/day × 5 days) in combination with caerulein (10^−8 ^M), as compared to the caerulein (10^−8 ^M) alone stimulation. The blots were stripped and probed with GAPDH to document equal protein loading. All presented results were obtained in 4 consecutive experiments and are representative for the observed phenomenon.

 The amount of Toll-like receptor 4 (TLR4) proteins in the pancreatic acinar cells at adult rats was determined in all examined samples (Figures 1 and 2). The ratio of TLR4/GAPDH protein level in the control group was 40.0 ± 0.2 and significantly dose-dependently increased in the group of rats treated at early period of life with 5, 10 or 15 mg/kg/day doses of LPS for 5 consecutive days. The highest abundance of protein was detected in the cell samples from animals treated with LPS at doses of 10 or 15 mg/kg/day and the ratio of TLR4/GAPDH reached 79.0 ± 0.4 and 98.0 ± 0.4, respectively (Figure 1).

Application of caerulein (10^−8 ^M) to the acinar cells significantly upregulated TLR4 protein level, as compared to the control group with ratio of TLR4/GAPDH 58.0 ± 0.3 after 5 hours of incubation (Figure 2).

Endotoxemia in the newborn rats induced by increasing doses of LPS (5, 10 or 15 mg/kg/day × 5 days) resulted in significant and dose-dependent increase of TLR4 protein level in the acini incubated with caerulein (10^−8 ^M) as compared to the group subjected to caerulein alone. The most significant increase was detected in the cells isolated from rats treated with LPS at doses of 10 or 15 mg/kg/day. The ratio of TLR4/GAPDH in these groups extended to 119.0 ± 0.4 and 130.0 ± 0.4, respectively (Figure 2).

**Figure 3:**
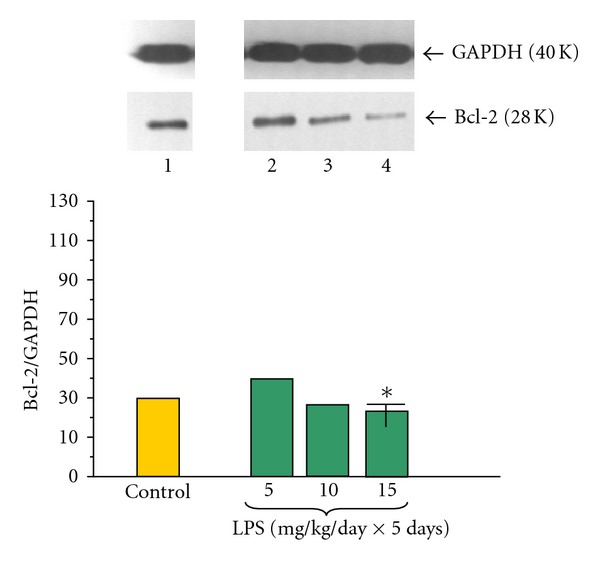
Western blot analysis of Bcl-2 protein level in the adult rat pancreatic acinar cells under basal conditions (lane 1), treated in the infant animals by lipopolysaccharide (*Escherichia coli*) at the doses of 5 mg/kg/day × 5 days (lane 2), 10 mg/kg/day × 5 days (lane 3), and 15 mg/kg/day × 5 days (lane 4) after 5 hours of incubation. Asterisk indicates significant (*P* < 0.05) change, as compared to the control group. The blots were stripped and probed with GAPDH to document equal protein loading. All presented results were obtained in 4 consecutive experiments and are representative for the observed phenomenon.

**Figure 4:**
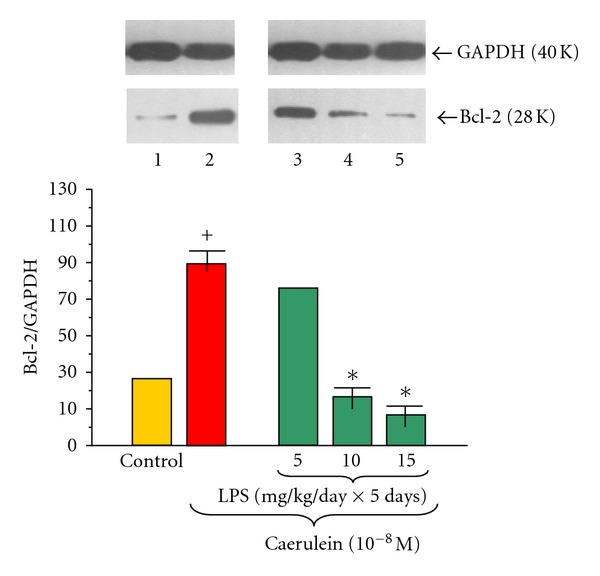
Western blot analysis of Bcl-2 protein level in the adult rat pancreatic acinar cells under basal conditions (lane 1), stimulated *in vitro* by caerulein at the dose of 10^−8 ^M (lane 2), treated in the infant animals by lipopolysaccharide (*Escherichia coli*) at the doses of 5 mg/kg/day × 5 days + caerulein 10^−8 ^M (lane 3), 10 mg/kg/day × 5 days + caerulein 10^−8 ^M (lane 4), and 15 mg/kg/day × 5 days + caerulein 10^−8 ^M (lane 5) after 5 hours of incubation. Cross indicates significant (*P* < 0.05) change, as compared to the control group. Asterisk indicates significant (*P* < 0.05) change, as compared to the value obtained from the rats treated by increasing doses of LPS (10 or 15 mg/kg/day × 5 days) in combination with caerulein (10^−8 ^M), as compared to the caerulein (10^−8 ^M) alone stimulation. The blots were stripped and probed with GAPDH to document equal protein loading. All presented results were obtained in 4 consecutive experiments and are representative for the observed phenomenon.

An antiapoptotic mitochondrial molecule Bcl-2 was detected in all examined samples of pancreatic acinar cells obtained from adult animals (Figures 3 and 4). The ratio of Bcl-2/GAPDH proteins in the control group was 29.00 ± 0.2 and significantly in dose-dependent manner decreased in the group of rats treated priorly with LPS at dose of 15 mg/kg/day × 5 days. The ratio of Bcl-2/GAPDH reached 22.0 ± 0.1 (Figure 3).

Application of caerulein (10^−8 ^M) to the acinar cells obtained from the control rats resulted in the significant upregulation of Bcl-2 protein level, as compared to the control, untreated with caerulein culture. The ratio of Bcl-2/GAPDH was 89.0 ± 0.3 after 5 hours of incubation (Figure 4).

Endotoxemia in the sucking animals caused by increasing doses of LPS (5, 10 or 15 mg/kg/day × 5 days) in significant and dose-dependent way downregulated Bcl-2 protein level in the pancreatic acini incubated with caerulein (10^−8 ^M) as compared to the caerulein-treated group alone. The strongest signal was detected in the acini gained from animals treated with LPS at doses of 10 or 15 mg/kg/day. The ratio in these groups of Bcl-2/GAPDH reached 17.0 ± 0.1 and 08.0 ± 0.05 respectively (Figure 4).

**Figure 5:**
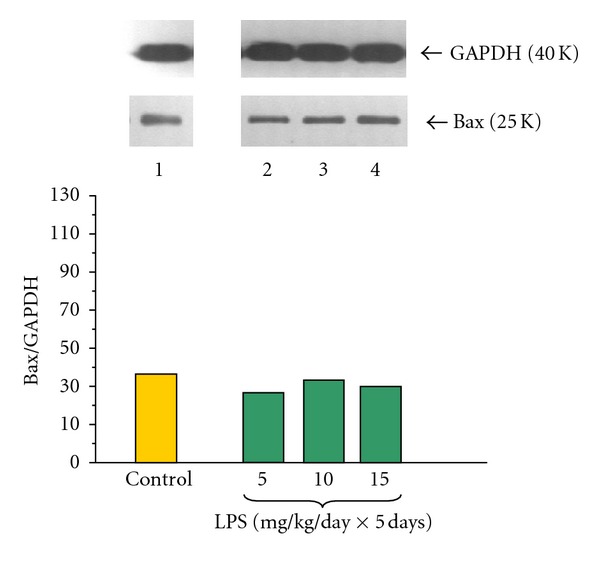
Western blot analysis of Bax protein level in the adult rat pancreatic acinar cells under basal conditions (lane 1), treated in the infant animals by lipopolysaccharide (*Escherichia coli*) at the doses of 5 mg/kg/day × 5 days (lane 2), 10 mg/kg/day × 5 days (lane 3), and 15 mg/kg/day × 5 days (lane 4) after 5 hours of incubation. The blots were stripped and probed with GAPDH to document equal protein loading. All presented results were obtained in 4 consecutive experiments and are representative of the observed phenomenon.

**Figure 6:**
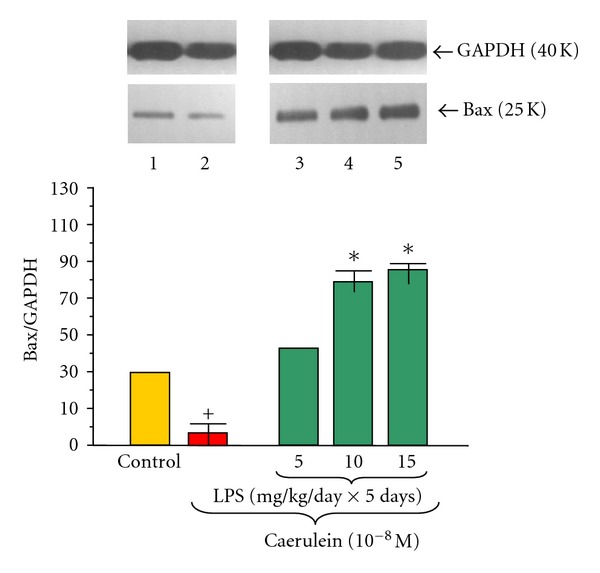
Western blot analysis of Bax protein level in the adult rat pancreatic acinar cells under basal conditions (lane 1), stimulated *in vitro* by caerulein at the dose of 10^−8 ^M (lane 2), treated in the infant animals by lipopolysaccharide (*Escherichia coli*) at the doses of 5 mg/kg/day × 5 days + caerulein 10^−8 ^M (lane 3), 10 mg/kg/day × 5 days + caerulein 10^−8 ^M (lane 4), and 15 mg/kg/day × 5 days + caerulein 10^−8 ^M (lane 5) after 5 hours of incubation. Cross indicates significant (*P* < 0.05) change, as compared to the control group. Asterisk indicates significant (*P* < 0.05) change, as compared to the value obtained from the rats treated by increasing doses of LPS (10 or 15 mg/kg/day × 5 days) in combination with caerulein (10^−8 ^M), as compared to the caerulein (10^−8 ^M) alone stimulation. The blots were stripped and probed with GAPDH to document equal protein loading. All presented results were obtained in 4 consecutive experiments and are representative of the observed phenomenon.

The proapoptotic mitochondrial Bax protein level in pancreatic acinar cells obtained from adult animals was detected in all examined samples (Figures 5 and 6). The ratio of Bax/GAPDH protein level in the control group was 38.00 ± 0.2 and did not change in the group treated priorly with increasing doses of LPS 5, 10 or 15 mg/kg/day × 5 days (Figure 5).

 Incubation of the pancreatic acinar cells with caerulein (10^−8 ^M) caused a significant decrease of Bax protein level, as compared to the untreated with caerulein control. The ratio of Bax/GAPDH protein in this group was 3.5 ± 0.05 after 5 hours of incubation (Figure 6).

**Figure 7:**
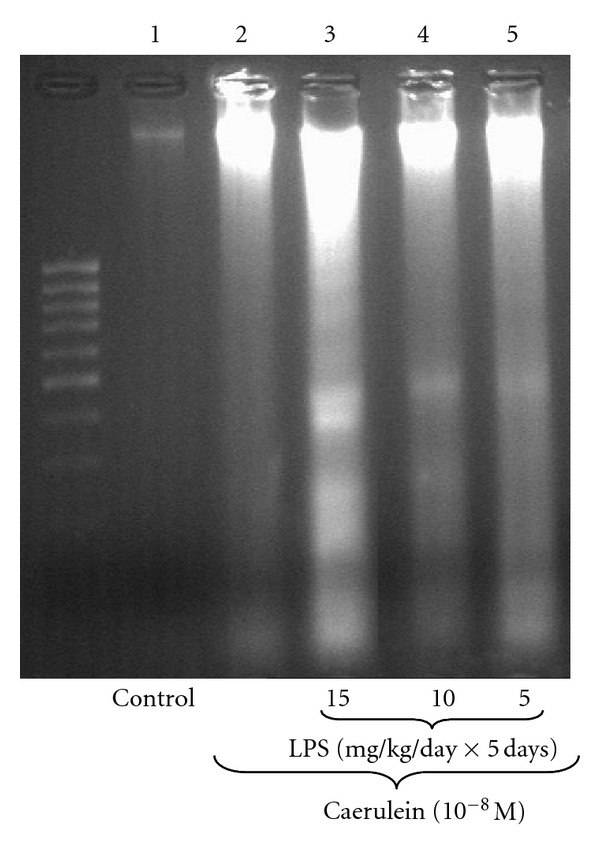
Analysis of DNA fragmentation pattern in the adult rat pancreatic acinar cells under basal conditions (lane 1), stimulated *in vitro* by caerulein at the dose of 10^−8 ^M (lane 2), treated in the infant animals by lipopolysaccharide (*Escherichia coli*) at the doses of 15 mg/kg/day × 5 days + caerulein 10^−8 ^M (lane 3), 10 mg/kg/day × 5 days + caerulein 10^−8 ^M (lane 4), and 5 mg/kg/day × 5 days + caerulein 10^−8 ^M (lane 5) after 5 hours of incubation. All presented results were obtained in 4 consecutive experiments and are representative of the observed phenomenon.

Endotoxemia in the newborn rats due to increasing doses of LPS (5, 10 or 15 mg/kg/day × 5 days) caused significant and dose-dependent increase of Bax protein level in the acini incubated with caerulein (10^−8 ^M). The strongest signals were detected in the animals treated with 10 or 15 mg/kg/day of LPS. In these groups the ratio of Bax/GAPDH reached 80.0 ± 0.4 and 87.0 ± 0.4, respectively (Figure 6). The data obtained in DNA fragmentation ladder assay correspond with the results of the analysis of apoptosis-related proteins, revealing the strongest pattern of DNA apoptotic damage in the cultures of pancreatic acini isolated from animals preconditioned with highest doses of LPS and subjected to caerulein (10^−8 ^M) stimulation (Figure 7 lane 3). In the control cultures of pancreatic acini and those subjected to caerulein stimulation without foregoing preconditioning with LPS no apoptosis-related DNA damage pattern was observed (Figure 7 lanes 1, 2).

**Figure 8:**
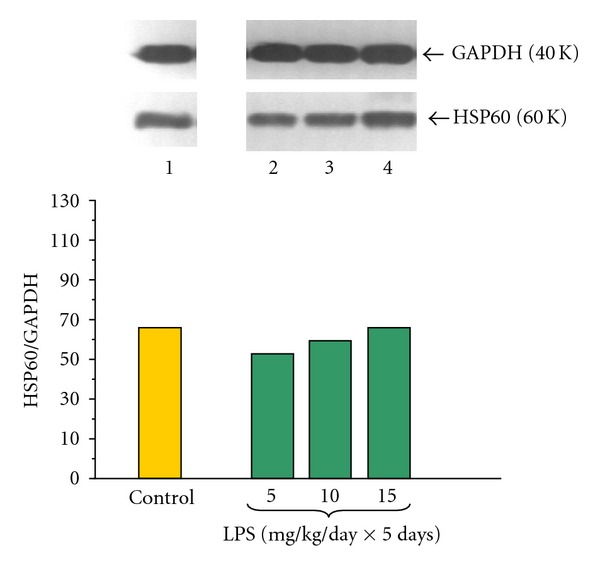
Western blot analysis of HSP60 protein level in the adult rat pancreatic acinar cells under basal conditions (lane 1), treated in the infant animals by lipopolysaccharide (*Escherichia coli*) at the doses of 5 mg/kg/day × 5 days (lane 2), 10 mg/kg/day × 5 days (lane 3) and 15 mg/kg/day × 5 days (lane 4) after 5 hours of incubation. The blots were stripped and probed with GAPDH to document equal protein loading. All presented results were obtained in 4 consecutive experiments and are representative of the observed phenomenon.

**Figure 9:**
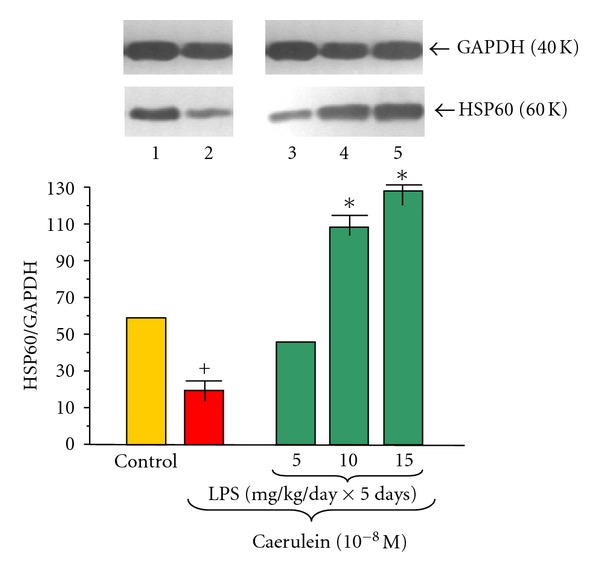
Western blot analysis of HSP60 protein level in the adult rat pancreatic acinar cells under basal conditions (lane 1), stimulated *in vitro* by caerulein at the dose of 10^−8 ^M (lane 2), treated in the infant animals by lipopolysaccharide (*Escherichia coli*) at the doses of 5 mg/kg/day × 5 days + caerulein 10^−8 ^M (lane 3), 10 mg/kg/day × 5 days + caerulein 10^−8 ^M (lane 4), and 15 mg/kg/day × 5 days + caerulein 10^−8 ^M (lane 5) after 5 hours of incubation. Cross indicates significant (*P* < 0.05) change, as compared to the control group. Asterisk indicates significant (*P* < 0.05) change, as compared to the value obtained from the rats treated by increasing doses of LPS (10 or 15 mg/kg/day × 5 days) in combination with caerulein (10^−8 ^M), as compared to the caerulein (10^−8 ^M) alone stimulation. The blots were stripped and probed with GAPDH to document equal protein loading. All presented results were obtained in 4 consecutive experiments and are representative of the observed phenomenon.

The HSP60 protein level was detected in all examined samples of pancreatic acinar cells obtained from adult animals (Figures 8 and 9). The ratio of HSP60/GAPDH protein level in the control group was 63.00 ± 0.3 and failed to change in the group treated at infancy by increasing doses of LPS 5, 10 or 15 mg/kg/day × 5 days (Figure 8).

Addition of caerulein (10^−8 ^M) to the pancreatic acinar cell culture obtained from untreated with LPS animals significantly decreased protein level of HSP60, as compared to the control group. The ratio of HSP60/GAPDH was 19.0 ± 0.03 after 5 hours of incubation (Figure 9).

 To the contrary, endotoxemia in the suckling rats produced significant increase of HSP60 protein level detected in the acini culture incubated with caerulein (10^−8 ^M), as compared to the cells subjected to caerulein alone (Figure 9). The most pronounced protein levels were detected in the cell culture obtained from animals treated with LPS at doses of 10 or 15 mg/kg. In these groups the distinct increase of HSP60/GAPDH ratio up to 109.0 ± 0.4 and 129.0 ± 0.5 was noticed, respectively (Figure 9).

**Figure 10:**
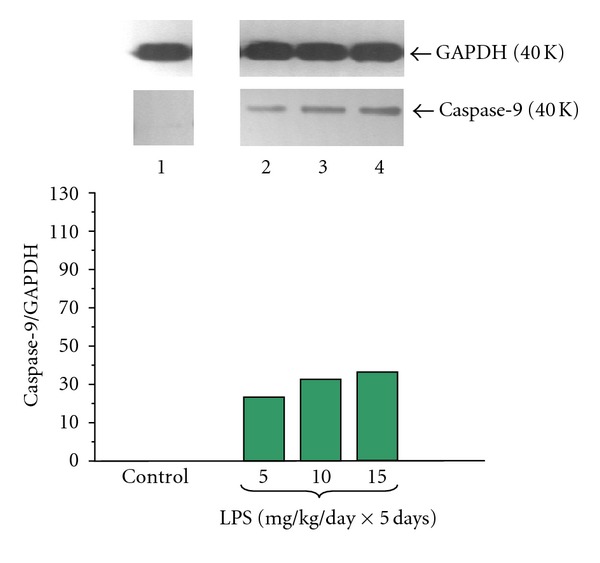
Western blot analysis of proapoptotic initiator caspase-9 protein level in the adult rat pancreatic acinar cells under basal conditions (lane 1), treated in the infant animals by lipopolysaccharide (*Escherichia coli*) at the doses of 5 mg/kg/day × 5 days (lane 2), 10 mg/kg/day × 5 days (lane 3), and 15 mg/kg/day × 5 days (lane 4) after 5 hours of incubation. The blots were stripped and probed with GAPDH to document equal protein loading. All presented results were obtained in 4 consecutive experiments and are representative of the observed phenomenon.

**Figure 11:**
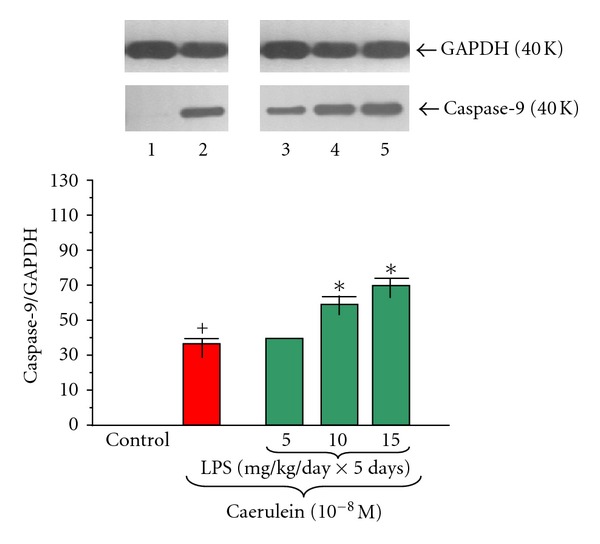
Western blot analysis of proapoptotic initiator caspase-9 protein level in the adult rat pancreatic acinar cells under basal conditions (lane 1), stimulated *in vitro* by caerulein at the dose of 10^−8 ^M (lane 2), treated in the infant animals by lipopolysaccharide (*Escherichia coli*) at the doses of 5 mg/kg/day × 5 days + caerulein 10^−8 ^M (lane 3), 10 mg/kg/day × 5 days + caerulein 10^−8 ^M (lane 4), and 15 mg/kg/day × 5 days + caerulein 10^−8 ^M (lane 5) after 5 hours of incubation. Cross indicates significant (*P* < 0.05) change, as compared to the control group. Asterisk indicates significant (*P* < 0.05) change, as compared to the value obtained from the rats treated by increasing doses of LPS (10 or 15 mg/kg/day × 5 days) in combination with caerulein (10^−8 ^M), as compared to the caerulein (10^−8 ^M) alone stimulation. The blots were stripped and probed with GAPDH to document equal protein loading. All presented results were obtained in 4 consecutive experiments and are representative of the observed phenomenon.

The proapoptotic initiator caspase-9 protein level was not detected in pancreatic acinar cells obtained from adult animals in untreated control cultures (Figures 10 and 11). Endotoxemia in the newborn rats due to increasing doses of LPS (5, 10 or 15 mg/kg/day × 5 days) was stimulatory factor for caspase-9 expression. The ratio of caspase-9/GAPDH reached 37.0 ± 0.2 (Figure 10).

Incubation of the pancreatic acinar cells with caerulein (10^−8 ^M) caused a significant increase of caspase-9 protein level. The ratio of caspase-9/GAPDH protein in this group was 40.0 ± 0.2 after 5 hours of incubation (Figure 11).

Foregoing endotoxemia evoked by increasing doses of LPS (5, 10 or 15 mg/kg/day × 5 days) caused marked and dose-dependent upregulation of caspase-9 protein level in the pancreatic acini incubated with caerulein (10^−8 ^M) as compared to the caerulein-treated group alone with the highest expression values detected in the acini cultures from animals treated with LPS at doses of 10 or 15 mg/kg/day. The ratio in these groups for caspase-9/GAPDH reached 62.0 ± 0.3 and 70.0 ± 0.3, respectively (Figure 11).

**Figure 12:**
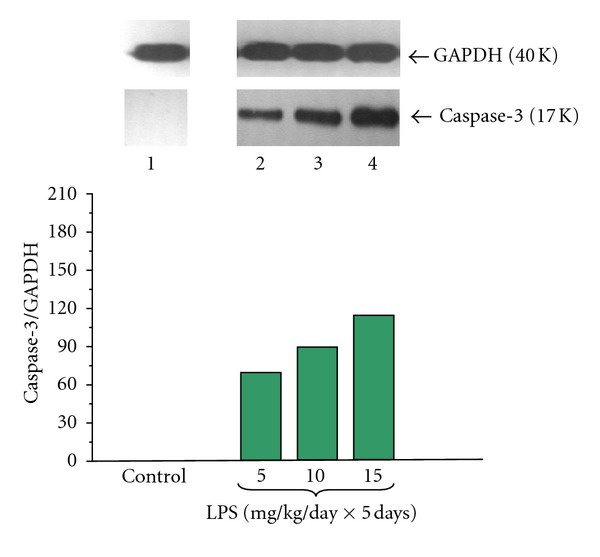
Western blot analysis of apoptosis executioner caspase-3 protein level in the adult rat pancreatic acinar cells under basal conditions (lane 1), treated in the infant animals by lipopolysaccharide (*Escherichia coli*) at the doses of 5 mg/kg/day × 5 days (lane 2), 10 mg/kg/day × 5 days (lane 3), and 15 mg/kg/day × 5 days (lane 4) after 5 hours of incubation. The blots were stripped and probed with GAPDH to document equal protein loading. All presented results were obtained in 4 consecutive experiments and are representative of the observed phenomenon.

**Figure 13:**
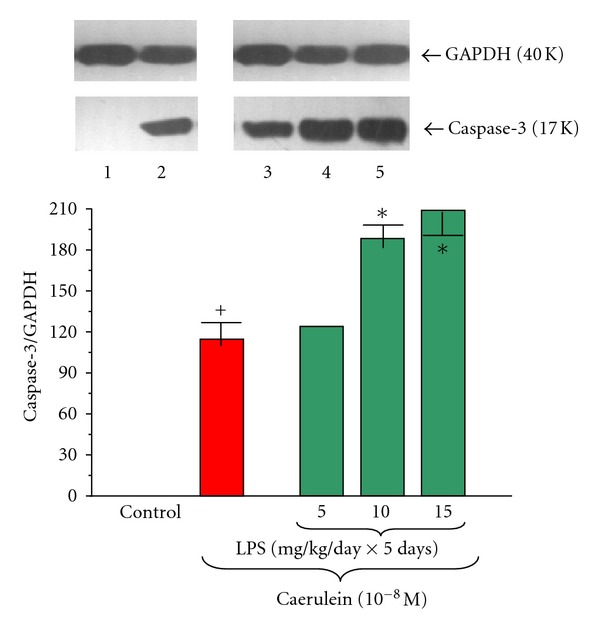
Western blot analysis of apoptosis executioner caspase-3 protein level in the adult rat pancreatic acinar cells under basal conditions (lane 1), stimulated *in vitro* by caerulein at the dose of 10^−8 ^M (lane 2), treated in the infant animals by lipopolysaccharide (*Escherichia coli*) at the doses of 5 mg/kg/day × 5 days + caerulein 10^−8 ^M (lane 3), 10 mg/kg/day × 5 days + caerulein 10^−8 ^M (lane 4), and 15 mg/kg/day × 5 days + caerulein 10^−8 ^M (lane 5) after 5 hours of incubation. Cross indicates significant (*P* < 0.05) change, as compared to the control group. Asterisk indicates significant (*P* < 0.05) change, as compared to the value obtained from the rats treated by increasing doses of LPS (10 or 15 mg/kg/day × 5 days) in combination with caerulein (10^−8 ^M), as compared to the caerulein (10^−8 ^M) alone stimulation. The blots were stripped and probed with GAPDH to document equal protein loading. All presented results were obtained in 4 consecutive experiments and are representative of the observed phenomenon.

Caspase-3 protein was not detected in pancreatic acinar cells obtained from adult animals in control samples (Figures 12 and 13). We have detected elevated level of caspase-3 protein in the acini isolated from animals subjected to the endotoxemia in the infancy due to increasing doses of LPS (5, 10 or 15 mg/kg/day × 5 days) with the ratio of caspase-3/GAPDH reaching 113.0 ± 0.4 (Figure 12).

Application of caerulein (10^−8 ^M) to the acinar cells significantly upregulated pro-apoptotic caspase-3 protein level. The ratio of caspase-3/GAPDH was at the level of 118.0 ± 0.4 after 5 hours of incubation (Figure 13).

Prior to LPS (5, 10 or 15 mg/kg/day × 5 days) endotoxemia resulted in significant and dose-dependent increase of caspase-3 protein level in the acini cultures incubated with caerulein (10^−8 ^M) as compared to the caerulein-treated group alone. The strongest signals were detected in the cell cultures obtained from the rats treated with LPS at the doses of 10 or 15 mg/kg/day. The ratio of caspase-3/GAPDH in these groups extended to 188.0 ± 0.4 and 209.0 ± 0.5, respectively (Figure 13).

## 4. Discussion

Acute pancreatitis (AP) is a pancreatic nonspecific inflammatory process resulting from the activation of many pathological mechanisms such as obstruction of pancreatic duct, acinar oversecretion, and pancreatic ischemia [21–24]. As the result of above processes the innate immune system is involved in development of inflammatory cascade. Toll-like receptors (TLRs) are suspected to trigger this reaction. It is currently thought that TLRs play an important role in the recognition of endogenous or exogenous antigens and in the initiation of signal transduction for inflammatory reaction during AP [36–38]. Therefore, investigating the tissue-specific expression of these receptors in the pancreas and exploring their role could be important for clarifying the pathogenesis of AP.

 In this study we demonstrate the presence of TLR4 on pancreatic acinar cells obtained from the adult rats. In the normal pancreas TLR4 are mainly localized in the epithelial (pancreatic duct epithelium) and endothelial tissue (arteries, veins, and microvascular endothelium) [39, 40]. Herein we have observed that TLR4 protein level in the pancreatic acini was dose-dependently increased in the animals, which have been treated in the early period of life with increasing doses of LPS (*Escherichia coli*). Since LPS has been identified as a ligand for TLR4, it is generally agreed that TLRs are upregulated under inflammatory conditions and downregulated by immunosuppression [2–4, 41–43]. In our study exposure of the pancreatic acinar cells to caerulein caused upregulation of TLR4 protein level. It was reported that these receptors have been rapidly upregulated during the early stage of rat caerulein-induced pancreatitis (CIP) and that might be associated with induction of apoptosis *via* the activation of both intrinsic and extrinsic apoptotic signaling pathways [39, 43, 44]. On the other hand, these receptors are downregulated in the late phase of severe acute pancreatitis (SAP) [45]. Moreover, TLR4 activity is associated with the increased apoptosis [46–51]. Treatment of acini with caerulein resulted in the dose-dependent increase of TLR4 protein level in the rats subjected to endotoxemia in the suckling period of life.

Acinar cell death and parenchymal necrosis is a major cause of severe complications and mortality in human pancreatitis [52, 53]. In AP acinar cells die through both necrosis and apoptosis. The severity of experimental pancreatitis correlates directly with the intensity of necrosis and, inversely, with apoptosis [53–55]. Bax and Bcl-2 family proteins are important regulators of cell apoptosis and their ratio determines the cell susceptibility to this process [56, 57]. Thus, elucidation of the mechanisms mediating acinar cells death in AP is important for understanding of the regulation of this disease and clinical relevance.

In present study we have demonstrate antiapoptotic mitochondrial molecule Bcl-2 in pancreatic acinar cells to be dose-dependently reduced in the group of rats treated at early period of life with highest dose of LPS. Application of caerulein to the acinar cells resulted in the upregulation of Bcl-2 and decrease of Bax protein levels, as compared to the control cells. AP has been shown to upregulate Bcl-2, whereas Bax mRNA was inhibited [58–60]. Our results revealed that in the pancreatic acinar cells obtained from the rats subjected in infancy to endotoxemia, caerulein caused dose-dependent: downregulation of antiapoptotic Bcl-2 and upregulation of pro-apoptotic Bax protein levels, as compared to the caerulein-treated cells alone, suggesting susceptibility of those cultures to apoptosis. Our assumption was confirmed with DNA fragmentation assay. Pancreatic acinar cells obtained from the rats subjected in infancy to endotoxemia and stimulated *in vitro* with caerulein manifested typical for apoptosis pattern of DNA damage.

 We have found HSP60 protein in pancreatic acinar cells obtained from adult animals and this confirmed the previous observations concerning the presence of HSP60 protein in the pancreas and in the pancreatic cell line; AR42J [30, 31, 61–63]. This expression of HSP60 in pancreatic tissues has been decreased with prolonged stimulation with LPS [64]. Ohashi et al. [14] claimed that TLR4 mediates HSP60 signaling, as a putative endogenous ligand of the TLR4 complex. We have observed that exposition of the acinar cells in culture to caerulein decreased protein level of HSP60, as compared to the control group. This is in agreement with Rakonczay et al. [65] who have shown that repeated injections of supramaximal doses of CCK to the rat could reduce pancreatic HSP60. Our previous data also demonstrated that caerulein stimulation is able to reduce mRNA signal for HSP60 in the AR42J cells [62, 63]. It is interesting that in the rat pancreas caerulein in time- and dose-dependent manner increases mRNA but paradoxically reduces protein level of rat pancreatic HSP60 [64, 66]. Endotoxemia in the suckling rats resulted in the increase of HSP60 protein level in pancreatic acinar cells, obtained from adult animals, and subjected to caerulein overstimulation. This is in agreement with previous report showing upregulation of pancreatic HSP60 after treatment with combination of caerulein and LPS [61]. On the other hand, different researchers showed time- and dose-dependent increases of mRNA that were followed by paradoxical reduction of protein level of rat pancreatic HSP60 after application of caerulein and LPS [64]. Our previous studies have shown that endotoxemia induced in the early period of life limits AP and reduces the pancreatic exocrine function in response to caerulein both *in vivo* and *in vitro* models at adult age [29–32]. It is possible that pancreatoprotective effects, like cytokine modulation, superoxide dismutase (SOD) activity, and decrease of pancreatic enzyme secretion, are related to the upregulation of HSP60 protein level in the pancreatic acini. Otaka et al. [18] and Rakonczay et al. [19, 20, 65] showed that an increase of HSP60 transcription and production and/or in conjunction with the cytokine modulation and free radical scavenger enzymes (e.g., SOD) activities could be important of “adaptive cytoprotection” in the AP. Le Gall and Bendayan [16], Li et al. [17] demonstrated hypothesis that the HSP60 would assist the proper folding and assembly of pancreatic secretory proteins and could also prevent their autoactivation before secretion and must be important for quality control and integrity of it.

In the present study we have not detected pro-apoptotic initiator caspase-9 and apoptosis executioner caspase-3 protein expression on pancreatic acinar cells obtained from the adult rats. However Gukovskaya et al. [67] and Mareninova et al. [68] have demonstrated the presence of active caspases-9 and -3 in the normal pancreatic tissue and pancreatic acini. We have shown that endotoxemia in the newborn rats, stimulated both caspases expression in acinar cells obtained from adult animals. This is in agreement with previous study showing that LPS treatment increased caspase-3 activity in the pancreas [69]. We have demonstrated that exposure of the acinar cells to caerulein (10^−8 ^M) upregulated pro-apoptotic caspase-9 and -3 protein level, as compared to the control group, what is in agreement with the observation of Gukovskaya et al. [67] and Mareninova et al. [68]. In experimental models of AP, acinar cells have been shown to die through necrosis and apoptosis [68]. We have found that endotoxemia in the sucking animals evoked by increasing doses of LPS caused dose-dependently upregulation of pro-apoptotic caspase-9 and executioner caspase-3 protein level in the pancreatic acini incubated with caerulein. Laine et al. [70] demonstrated that the LPS causes release of pancreatic phospholipase A_2_ (PLA_2_) into blood, its activation in pancreatic tissue and apoptosis of acinar cells. Kimura et al. [71] showed that LPS pretreatment increased remarkably the incidence of acinar cells apoptosis in AP. These results suggest that the pathological features of this disease might be modified by the presence of nonfatal endotoxemia through the induction of acinar cells apoptosis.

 In conclusion, our data indicate that exposure of the infant rats to LPS promoted the induction of HSP60 *via* TLR4 in their adult life and, in turn, activated Bax/Bcl-2 and caspase-9 and -3. It is likely that this process could take a part in the LPS-induced protection of the pancreatic tissue against acute damage produced by caerulein overstimulation.
